# Interrelations Between Temporal and Spatial Cognition: The Role of Modality-Specific Processing

**DOI:** 10.3389/fpsyg.2018.02609

**Published:** 2018-12-21

**Authors:** Jonna Loeffler, Rouwen Cañal-Bruland, Anna Schroeger, J. Walter Tolentino-Castro, Markus Raab

**Affiliations:** ^1^Department of Performance Psychology, Institute of Psychology, German Sport University Cologne, Cologne, Germany; ^2^Institute of Sport Science, Friedrich-Schiller-University Jena, Jena, Germany; ^3^School of Applied Sciences, London South Bank University, London, United Kingdom

**Keywords:** time-space mapping, asymmetry hypothesis, symmetry hypothesis, conceptual metaphor theory, a theory of magnitude, spatial representation, temporal representation

## Abstract

Temporal and spatial representations are not independent of each other. Two conflicting theories provide alternative hypotheses concerning the specific interrelations between temporal and spatial representations. The asymmetry hypothesis (based on the conceptual metaphor theory, Lakoff and Johnson, 1980) predicts that temporal and spatial representations are *asymmetrically* interrelated such that spatial representations have a stronger impact on temporal representations than vice versa. In contrast, the symmetry hypothesis (based on a theory of magnitude, Walsh, 2003) predicts that temporal and spatial representations are *symmetrically* interrelated. Both theoretical approaches have received empirical support. From an embodied cognition perspective, we argue that taking sensorimotor processes into account may be a promising steppingstone to explain the contradictory findings. Notably, different modalities are differently sensitive to the processing of time and space. For instance, auditory information processing is more sensitive to temporal than spatial information, whereas visual information processing is more sensitive to spatial than temporal information. Consequently, we hypothesized that different sensorimotor tasks addressing different modalities may account for the contradictory findings. To test this, we critically reviewed relevant literature to examine which modalities were addressed in time-space mapping studies. Results indicate that the majority of the studies supporting the asymmetry hypothesis applied visual tasks for both temporal and spatial representations. Studies supporting the symmetry hypothesis applied mainly auditory tasks for the temporal domain, but visual tasks for the spatial domain. We conclude that the use of different tasks addressing different modalities may be the primary reason for (a)symmetric effects of space on time, instead of a genuine (a)symmetric mapping.

## Introduction

For complex human behavior, including sensorimotor actions such as catching a ball, precise representations of time and space are of utmost importance (e.g., Rosenbaum et al., 2012). For instance, in movement-related tasks the anticipation of duration (= time) and distance (= space) influences manifold decisions about how to act such as when deciding whether to cross the street or stop walking (Zito et al., 2015), whether to accelerate or slow down when trying to catch a ball (Postma et al., 2017), or whether to wait for the elevator or take the stairs (Wittmann, 2014). In order to predict environmental demands and to plan actions, an actor has to constantly and adequately represent temporal and spatial information (Postma et al., 2017). For example, the looming sound of an approaching car helps a pedestrian to estimate its speed and moment of passing and thus to adjust movements and avoid a collision. This is the very reason why e-cars, which typically do not generate sounds, are considered more dangerous for pedestrians than normal cars. As a consequence, a law in the US requires all newly manufactured e-cars to produce auditory noise when driving. Though it is well-known that interrelations between temporal and spatial representations are essential for human functioning, the mechanisms underlying these interrelations are far from being well understood.

When reviewing the literature that addresses the (a)symmetry of time and space, it is evident that there is no consensus about the intimate links between temporal and spatial representations (Winter et al., 2015). Two influential and currently debated hypotheses are the *asymmetry hypothesis*, which is based on the conceptual metaphor theory (= CMT, Lakoff and Johnson, 1980; Boroditsky, 2000) and the *symmetry hypothesis*, which is based on a theory of magnitude (= ATOM, e.g., Walsh, 2003). Both assume different relationships between temporal and spatial representations and, as a consequence make divergent claims about how time-space mappings modulate movements. Notwithstanding the divergent predictions, both hypotheses received robust empirical support (e.g., Boroditsky, 2000; Merritt et al., 2010; Agrillo and Piffer, 2012; Bottini and Casasanto, 2013; Hyde et al., 2013; Skagerlund and Träff, 2014; Xue et al., 2014; Coull et al., 2015; Skagerlund et al., 2016, see Tables 1, 2 for an overview). The question arises as to how it is possible that two contradicting hypotheses seem to both have received robust empirical support? In search of the mechanisms that cause the contradictory findings, it is important to realize that the different modalities are differently sensitive to the processing of time and space. Consequently, we hypothesized that different sensorimotor tasks addressing different modalities may account for the contradictory findings. Based on this assumption, in this mini-review we critically review relevant literature to examine which modalities were addressed in time-space mapping studies.

**Table 1:** Studies supporting the conceptual metaphor theory and therefore an *asymmetric* time-space mapping.

**Study**	**Participants**	**Temporal and spatial tasks: modalities**	**Independent variables**	**Dependent variables**	**Main finding**
Boroditsky, 2000	Exp. 1: *N* = 98 Exp. 2: *N* = 302 Exp. 3: *N* = 53	Space: visual Time: visual	Exp. 1–3: Temporal and spatial prime questions to prime either an ego-moving or object-moving frame of reference	Consistent response between prime and target questions (%); confidence score	Asymmetric time-space mapping, evidence for conceptual metaphor theory
Casasanto and Boroditsky, 2008	Exp. 1-3: *N = 9* Exp. 4: *N = 16* Exp. 5: *N = 10* Exp. 6: *N = 19*	Space: visual Time: visual and auditive	Duration/ spatial displacement of stimuli (growing lines/ moving dot) presented on a computer screen	Temporal or spatial judgment (Cross-dimensional interference effects; effect of distance on time estimation/effect of time on distance estimation)	Behavioral asymmetry: we rely on spatial information to make temporal estimates (particularly when space and time are conflicted in motion); not vice versa -> not only linguistic, here also nonlinguistic (representations for estimation)
Casasanto et al., 2010	N = 99 native Greek-speaking children	Space: visual Time: visual	Presentation of “racing snails” with congruent/incongruent traveled distance (spatial) and duration (temporal), duration/distance tasks without spatial/temporal interference	Temporal or spatial judgement (cross-dimensional interference tasks), distance or duration judgment (non-interference tasks)	Space and time related asymmetrically, evidence for conceptual metaphor theory (children can ignore irrelevant temporal information when making judgments about space, but have difficulty ignoring spatial information when making judgments about time)
Merritt et al., 2010	2 rhesus monkeys, 16 adult humans	Space: visual Time: visual	Presentation of lines with congruent/incongruent length (spatial) and duration (temporal)	Temporal or spatial judgments, influence of irrelevant dimension (space or time) on relevant dimension (space or time)	In humans: Asymmetrical time-space interactions predicted by conceptual metaphor theory; In monkeys: Symmetrical time-space interactions
Bottini and Casasanto, 2013	*N* = 56 native Dutch-speaking and Portuguese-speaking children (4–10 years old)	Space: visual Time: visual	Presentation of “racing snails” with congruent/incongruent traveled distance (spatial) and duration (temporal), duration/distance tasks without spatial/temporal interference	Temporal or spatial judgment (cross-dimensional interference tasks), distance or duration judgment (non-interference tasks)	Space and time related asymmetrically, evidence for conceptual metaphor theory (children can ignore irrelevant temporal information when making judgments about space, but have difficulty ignoring spatial information when making judgments about time)
Xue et al., 2014	*N* = 24 (Chinese)	Space: visual Time: visual	Chinese and English sentences, (correct/incorrect) containing temporal ordering and spatial sequencing	Acceptability ratios, ERPs	Neural representations during temporal sequencing and spatial ordering in both languages different, time-spatial relationship is asymmetric, evidence for conceptual metaphor theory
Coull et al., 2015	*N* = 16	Space: visual Time: visual	Duration or distance of dynamic trajectory of a moving dot (or static line stimulus, control condition)	fMRI (comparison of the accumulation of information in temporal vs. spatial domains)	Shared magnitude system, but time-space asymmetry
Zito et al., 2015	N = 36 (18 old and 18 young participants)	Space: visual Time: visual	Virtual reality with slow traffic condition (cars driving 30km/h) vs. a fast traffic condition (cars driving 50 km/h)	Street crossing behavior (temporal or spatial judgement), eye and head movements, non-parametric tests	Both groups paid more attention to space (distance of oncoming cars) than to time (speed of the cars) -> asymmetric; younger pedestrians behaved in a more secure manner while crossing a street (as compared to old people)

**Table 2:** Studies supporting the theory of magnitude, and therefore a *symmetric* time-space mapping.

**Study**	**Participants**	**Temporal and spatial tasks: modalities**	**Independent variables**	**Dependent variables**	**Main finding**
Agrillo and Piffer, 2012	*N* = 27 (13 professional musicians, 14 non-musicians)	Space: visual Time: auditory	Temporal (which of two tones lasted longer), spatial (which line was longer), numerical discrimination (which group of dots was more numerous) tasks	Judgment ratio, accuracy	Musicians (= experts in temporal discrimination) were not only better in temporal discrimination, but also in spatial discrimination, evidence for a shared magnitude system
Hyde et al., 2013	*N* = 32 (five-month old infants)	Space: visual Time: auditory	Relationally congruent/incongruent audio-visual length-time pairings	ERPs	Preverbal infants show incongruent effects when temporal and spatial magnitude do not match, evidence for a shared magnitude system
Skagerlund and Träff, 2014	*N* = 82	Space: visual Time: visual	Magnitude processing tasks: Space, time and number processing, screening tests, domain-general cognitive abilities	Response times	Children with dyscalculia displayed difficulties across time, space, and number magnitude processing tasks, evidence for a shared magnitude system
Cai and Connell, 2015	*N* = 32	Space: haptic Time: auditory	Touching (without seeing) physical sticks while listening to a congruent/incongruent auditory note	Reproducing length and duration of the presented stick/auditory note	Space-time mapping depends on the perceptual acuity of the modality used to perceive space, evidence for a shared magnitude system
Skagerlund et al., 2016	*N* = 24	Space: visual Time: visual	Time, space, and number discrimination tasks	Accuracy, response times, fMRI	Overlapping neural substrates across multiple magnitude dimensions, evidence for a shared magnitude system

Focusing on the role of modalities during the processing of temporal and spatial information, it should be considered that auditory information processing shows enhanced sensitivity to temporal information but lower sensitivity to spatial information (e.g., O'Connor and Hermelin, 1972; Recanzone, 2009). By contrast, visual information processing shows higher sensitivity to spatial information but lower sensitivity to temporal information (e.g., O'Connor and Hermelin, 1972; Recanzone, 2009). However, in audio-visual conditions, people tend to use the modality with the highest informational value to solve the task (e.g., Zhou et al., 2007). To illustrate, people are better in deducing spatial information regarding an approaching car when presented with information visually compared to being presented with auditory information. Therefore, when deducing temporal and spatial information from an approaching car, vision is our dominating system and thereby relatively impervious to distortion (Keshavarz et al., 2017). By contrast, in foggy environments, when the car is almost invisible, auditory information becomes more important. This relative importance of modality information depending on the informational value becomes also apparent when individual capacities are considered, as for example in blind subjects playing tennis with rattling balls. Further empirical evidence for the strong dependence on modality-related task characteristics is supported by illusion effects in which one modality dominates the perception of a multisensory object or event (Radeau and Bertelson, 1974). These illusion effects seem to be largely driven by the sensory modality that has the highest informational value for solving the task (for a review, see Recanzone, 2009).

In sum, the different sensitivities of different modalities to temporal and spatial information might moderate the empirical results. Because auditory information processing is more sensitive to temporal than spatial information and visual information processing is more sensitive to spatial than temporal information, it is reasonable to argue that different sensorimotor tasks may address auditory and visual information processing to different degrees. If true, then it can be hypothesized that different tasks addressing mainly one modality might cause the contradictory results with respect to the (a)symmetry of temporal and spatial representations. To test this, here we review the relevant literature to examine which modalities were addressed in studies that examined interrelations between temporal and spatial representations, supporting either the asymmetry or the symmetry hypothesis.

## Theoretical Background: CMT vs. ATOM

According to the asymmetry hypothesis, spatial representations grounded in movement have a stronger impact on temporal representations than vice versa. The asymmetry hypothesis is based on the conceptual metaphor theory (= CMT), which assumes that the neural system characterizing concrete sensorimotor experience has more inferential connections and therefore a greater inferential capacity than the neural system characterizing abstract thoughts (Lakoff and Johnson, 1980; Boroditsky, 2000). It follows that the abstract representation of time tends to be asymmetrically dependent on the more concrete representation of space. This asymmetric relationship between time and space, which is at the core of the asymmetry hypothesis, was originally supported by the analysis of metaphorical language (Clark, 1973; Lakoff and Johnson, 2003): When we talk about time, we mainly use spatial terms that often include movement (e.g., “The weekend is getting closer,” “The birthday is behind me”). Only rarely do we use temporal terms to talk about space (“I am five minutes from the central station,” see Cai and Connell, 2015). A number of studies have provided evidence that these linguistic expressions reflect a deeper, asymmetric conceptual link between time and space (Boroditsky, 2000; Merritt et al., 2010; Bottini and Casasanto, 2013; Xue et al., 2014; Coull et al., 2015), with concurrent spatial information affecting time judgments (e.g., duration) to a greater extent than concurrent temporal information affecting spatial judgments (e.g., length). Taken together, a plethora of studies seems to support the *asymmetry hypothesis* and its assumption that spatial representations have a stronger impact on temporal representations than vice versa.

In contrast, according to the symmetry hypothesis, which is based on a theory of magnitude (= ATOM), it is assumed that time and space are processed by a shared analog magnitude system (Walsh, 2003). In keeping with ATOM, temporal and spatial representations are processed in a common neural substrate and share representational and attentional resources (e.g., Walsh, 2003). The shared system for magnitudes of time and space (and numbers) explains compatibility effects without specifying any directionality of the effects. If space and time are both represented by the same general-purpose analog magnitude metric, there is no a-priori reason to posit that representations in one domain should depend asymmetrically on representations in the other. Empirical evidence for ATOM is provided by studies showing, for example, that expertise in temporal tasks (e.g., musicians) shows a positive transfer to spatial tasks (Agrillo and Piffer, 2012), or that overlapping neural substrates are active across temporal and spatial magnitude tasks (Skagerlund et al., 2016). By now, there is considerable empirical evidence for the *symmetry hypothesis* that space and time share the same basic spatio-temporal metrics and thereby equally influence each other (Walsh, 2003; Agrillo and Piffer, 2012; Hyde et al., 2013; Skagerlund and Träff, 2014; Cai and Connell, 2015; Skagerlund et al., 2016).

To summarize, on the one hand, there is empirical evidence for the asymmetry hypothesis and its main assumption that time and space remain two separate representational systems, with spatial representations being paramount in shaping our understanding of time, whereas temporal representations have less relevance when making spatial judgments (Boroditsky, 2000; Merritt et al., 2010; Bottini and Casasanto, 2013; Xue et al., 2014; Coull et al., 2015). On the other hand, there is empirical evidence to support the symmetry hypothesis that time and space share a common representational system, and hence, are symmetrically interrelated (Agrillo and Piffer, 2012; Hyde et al., 2013; Skagerlund and Träff, 2014; Cai and Connell, 2015; Skagerlund et al., 2016).

## Scope of Mini-Review: Selection Criteria

The aim of this short review is to critically assess the literature supporting either the asymmetry hypothesis (Lakoff and Johnson, 1980; CMT, Boroditsky, 2000) or the symmetry hypothesis (ATOM, Walsh, 2003) with a special focus on the question whether different tasks addressing different modalities may be the primary reason for (a)symmetric effects of space on time, instead of a genuine (a)symmetric mapping. To this end, we assessed whether the temporal and spatial tasks in the studies addressed the visual and/or auditory modality. As both hypotheses have variants that refer to the same theory but use different wording (e.g., “*metaphorical* mapping,” “*magnitude* system”), the literature search was based on the core words for each theorical background (“metaphor,” “magnitude”). Therefore, the authors performed two database searches (Web of Science, 24th of March 2018) using the terms (a) “metaphor^*^,” “time” or “temporal,” and “space” or “spatial,” and (b) “magnitude^*^,” “time” OR “temporal,” and “space” OR “spatial.” Papers with these three terms in the title were included. The search resulted in (a) 36 and (b) 40 results. To extend and validate the search results, the authors performed an additional database search using the terms: “time-space” or “space-time” and “asymmetr^*^ mapping,” or “symmetr^*^ mapping.” The search resulted in only four hits, of which one was in favor of the symmetry hypothesis. This article was therefore added to b). Two were off-topic and the fourth article was non-empirical and therefore not included.

From the list of papers resulting from the literature search, we selected only empirical studies that focused on time as well as on space (e.g., some studies focused on temporal metaphors without addressing the time-space (a)symmetry or others were completely off-topic). Although important for the understanding of the interrelations of time and space, the following review makes no statements about accounts concerning the processing stage in which the interrelation might occur (encoding, memory interference, retrieval) or about other possible moderators or modulators (e.g., Wang and Cai, 2017). Furthermore, neural correlates of spatial and temporal representations are not discussed within the scope of this mini-review. In addition, based on suggestions by an anonymous reviewer, two further studies important in the context of temporal and spatial representations were added (Casasanto and Boroditsky, 2008 and Casasanto et al., 2010). In the end, 16 studies were included in the analysis (see Tables 1–3). These 16 studies will be summarized with a special focus on the modality of the applied tasks.

**Table 3:** Studies examining temporal and spatial representations, but suggesting neither an asymmetric or symmetric time-space mapping.

**Study**	**Participants**	**Temporal and spatial tasks: modalities**	**Independent variables**	**Dependent variables**	**Main finding**
Yates et al., 2012	Exp. 1: *N* = 16 Exp. 2: *N* = 16	Space: visual Time: visual	Small and large squares differing in duration	Exp. 1: Duration judgment (longer/shorter than previous stimuli) Exp. 2: Duration judgment (same/different than previous stimuli)	Larger stimuli were judged—though not necessarily perceived—as *shorter* in duration
Rousselle et al., 2013	20 patients with Williams Syndrome 40 typically developing children	Space: visual Time: auditory	Temporal (which of two tones lasted longer), spatial (which line was longer), and numerical (which group of dots was more numerous) discrimination tasks, visuo-spatial task	Working memory of space, judgment ratio of time and space	The number processing difficulty of patients with Williams Syndrome was related to difficulties in visuo-spatial magnitude processing; auditory processing was not related to number processing difficulty
Cai and Connell, 2016	Exp. 1: *N* = 26 Exp. 2: *N* = 18	Space: visual Time: visual	Exp. 1: Visual flicker and spatial distance at either encoding (Exp. 1a) or reproduction (Exp. 1b) stage Exp. 2: Replication of Exp. 1, but with a within-subject design	Exp. 1a: Participants reproduced the stimulus duration while a neutral visual stimulus appeared onscreen Exp. 1b: Participants reproduced the stimulus duration while the visual flicker or spatial distance stimulus appeared onscreen. Exp. 2: Same as in Exp. 1	Exp. 1: Visual flicker affected time perception at both encoding and reproduction stages, whereas spatial distance affected time perception at the encoding stage only Exp. 2: Replication of Exp. 1

## Asymmetry vs. Symmetry Hypothesis: A Modality-Specific Analysis

Results indicate that most studies in favor of an *asymmetric* time-space mapping (Table 1) used visual tasks for both temporal and spatial representations (Boroditsky, 2000; Casasanto et al., 2010; Merritt et al., 2010; Bottini and Casasanto, 2013; Xue et al., 2014; Coull et al., 2015; Zito et al., 2015). Only one study (Casasanto and Boroditsky, 2008) included an audiovisual task but only for temporal judgments. Tasks applied were, for example, duration and distance judgments (Bottini and Casasanto, 2013) or ambiguous temporal and spatial questions (Boroditsky, 2000).

All reviewed studies in favor of a *symmetric* time-space mapping (Table 2, Agrillo and Piffer, 2012; Hyde et al., 2013; Skagerlund and Träff, 2014; Skagerlund et al., 2016) used visual tasks for the spatial domain only (except for one study that applied haptic tasks, Cai and Connell, 2015). With respect to the temporal domain, most of the studies in favor of the symmetry hypothesis applied an auditory task to measure temporal representations. Tasks included, for instance, temporal (e.g., which of two tones lasted longer) and spatial (e.g., which of two lines was longer) discrimination tasks (Hyde et al., 2013), or incongruent vs. congruent audio-visual length-time pairings (Agrillo and Piffer, 2012). One study (Skagerlund and Träff, 2014) used a visual task for measuring temporal performance.

The results of three studies support neither a symmetric nor asymmetric time-space mapping (Table 3; Yates et al., 2012; Rousselle et al., 2013; Cai and Connell, 2016). These reviewed studies applied visual tasks (except one study that applied an auditory task for the temporal domain, Rousselle et al., 2013), consisting of, for example, temporal and spatial distance judgments tasks (Cai and Connell, 2016) or temporal and spatial discrimination tasks (Rousselle et al., 2013). Importantly, Yates et al. (2012) investigated whether the found interrelations between time and space are due to affected representations or whether they are influenced by a decisional bias. As they found a reversed effect of space on time when changing the comparative task to an equality judgement they concluded that the given response requirements might affect the interaction between space and time as well. These findings neither support ATOM nor CMT. Therefore, the study was categorized to Table 3.

Furthermore, we decided not to list Cai and Connell (2016) in Table 2, supporting the symmetry hypothesis based on ATOM, but in Table 3 as the authors did not investigate the bidirectionality of the relationship between temporal and spatial representations. Only the influence of space on time was examined and therefore no conclusion concerning the (a)symmetry was drawn. Note though that Cai and Connell (2016) interpreted their results as being favorable toward the internal clock model (Gibbon et al., 1984) which is based on ATOM.

Finally, Rousselle et al. (2013) failed to support the symmetry hypothesis in their study. They showed a relationship between the magnitude perception of numbers and space but no association to time perception. Hence, their results support neither of the two theories and were also included in Table 3.

## Discussion and Conclusions

Based on the evaluation of 16 studies that were included in this short review, the results seem to provide initial support for the assumption that the use of different tasks addressing different modalities may account for (a)symmetric effects of space on time. In fact, the studies supporting the symmetry hypothesis predominantly used auditory tasks (and not visual tasks) when compared to studies supporting the asymmetry hypothesis. Given the discrepancy in the theoretical interpretation of the corresponding findings we suggest that (task-dependent) modality-specific processing plays a significant role for interrelations between temporal and spatial representations. Therefore, taking modality-specific processing into account when putting the conflicting hypotheses to test seems mandatory in order to shed light on the mechanisms underlying the interrelation between temporal and spatial representations.

Based on our assessment, it seems justified to argue that the studies in favor for either asymmetry or symmetry could easily be re-interpreted. For example, in Coull et al. (2015) asymmetry experiment it is apparent that the spatial and the temporal information were both provided by visual information. If we consider that visual information processing shows higher sensitivity to spatial information yet lower sensitivity to temporal information (e.g., Recanzone, 2009), the observed asymmetry could be based on the different informational values of vision and audition with respect to spatial and temporal information. In other words, when only visual information (but no auditory information) was provided, the reported asymmetry between space and time may hinge on that fact that the task was purely visual, and hence had a higher informational value for space than for time. In this context, Wang and Cai (2017), for instance, suggest that the cross-dimensional magnitude interaction depends on the amount of representational noise. If the rated construct is noisier and thus less reliable, it is more likely to be influenced by other magnitudes. Cai et al. (2018) therefore provide a Bayesian interference model to explain the findings.

Although the literature indicates that modality-specificity might matter when examining temporal and spatial representations, results were not distinctly clear: Some studies showed evidence for a symmetric time-space mapping, even though they applied a visual task to measure temporal representations. This pattern might be caused by the fact that modality-sensitivity is not the only factor influencing time-space mappings. Sticking with the assumption that there may be no genuine time-space (a)symmetry, there are some other factors—besides modality-specificity—that likely have an impact on the (a)symmetry of time and space. Other potential moderators could be, for example, the task automaticity/familiarity and response properties that cause decisional bias (Yates et al., 2012). In addition, the participant's age could be a moderator given that temporal vision matures more rapidly than spatial vision during childhood (Ellemberg et al., 1999). Furthermore, it is still under debate at which stage of processing the interference between time and space occurs (encoding, memory interference, retrieval, e.g., Cai et al., 2018). Cross-dimensional relations might differ depending on the different stages of processing and provide avenues for future research.

Although it seems challenging to dissociate cross-dimensional interactions, future studies might benefit from applying tasks that genuinely require both a balanced representation of time *and* space. Potential tasks resembling a more balanced representation of time and space include movement tasks such as catching a ball, as temporal and spatial representations play an analogous role for the execution of such movements. Further, recent evidence shows the importance of auditory information, additional to visual information, in anticipation tasks of moving stimuli (e.g., the landing location of a tennis ball, Cañal-Bruland et al., 2018). A crucial role of movements in interrelations of temporal and spatial representations is additionally supported by the fact that the processing of such quantities overlaps in parietal brain regions associated with action control (Bueti and Walsh, 2009). It is assumed that we learn associations occurring across different magnitude domains by moving in our environment. For example, catching a ball that was thrown from far away requires slower running speed than catching a ball that was thrown from a nearer distance (assuming that the balls were thrown with the same speeds and one was trying to catch at the same interception location). Therefore, in future studies, a task that genuinely contains movement (i.e., catching a ball), and provides visual as well as auditory information, might be beneficial to investigate the mechanisms that drive time-space mappings. Surely, future empirical research including movement in the task and taking potential moderators (e.g., modality-specificity, task automaticity, age) into account is needed to confirm or reject our assumptions.

A potential limitation of our short review is that it is quite likely that not all studies scrutinizing time-space mappings were covered by our literature search. One evident reason is that different terms and wording have been used in different studies. We cannot rule out that some studies, for example, provide evidence for symmetric time-space mappings without naming it time-space mapping or mentioning ATOM.

In summary, our literature review highlighted that seemingly contradictory claims could be bridged if cross-dimensional magnitude interactions between temporal and spatial representations were considered. It follows that previous experiments that examined only one modality may have limited success to specify the (a)symmetry of temporal and spatial representations and hence do not provide a proper test to tease the conflicting hypotheses apart. Consequently, a systematic manipulation of the relative contributions of different modalities to executing task-appropriate solutions in both the space-sensitive visual domain and the time-sensitive auditory domain seems necessary. Taking a task such as catching a ball as a testbed might be a promising approach to draw conclusions about the (a)symmetry of temporal and spatial representations.

## Author Contributions

All authors listed have made a substantial, direct and intellectual contribution to the work, and approved it for publication.

### Conflict of Interest Statement

The authors declare that the research was conducted in the absence of any commercial or financial relationships that could be construed as a potential conflict of interest.
